# Understanding the factors affecting self-management of COPD from the perspectives of healthcare practitioners: a qualitative study

**DOI:** 10.1038/s41533-017-0054-6

**Published:** 2017-09-18

**Authors:** Oladapo J. Ogunbayo, Sian Russell, James J. Newham, Karen Heslop-Marshall, Paul Netts, Barbara Hanratty, Eileen Kaner

**Affiliations:** 1 Institute of Health and Society, Newcastle University, Baddiley-Clark Building, Richardson Road, Newcastle upon Tyne, NE2 4AX UK; 2 NHS Newcastle Gateshead Clinical Commissioning Group, Newcastle upon Tyne, UK

## Abstract

Self-management is recognised as an essential criteria for the provision of high quality care for chronic obstructive pulmonary disease (COPD). The management of COPD is usually delivered by a wide range of healthcare practitioners. This study aimed to understand the factors affecting self-management of COPD from the perspectives of the different multidisciplinary healthcare teams involved in COPD care. Semi-structured interviews were conducted with participants from primary care, specialist respiratory and pulmonary rehabilitation (PR) teams. Purposive sampling and snowballing were employed in participant recruitment. All interviews were audio-recorded and transcribed verbatim and data were analysed thematically. A total of 20 participants (eight primary care practitioners, seven respiratory specialists and five PR practitioners) were interviewed until data saturation was reached. Participants identified a range of complex and interrelated factors affecting COPD self-management that were grouped into three broad categories—patient, practitioner and organisational/system-level factors. Patient-level factors were predominantly considered as barriers, with COPD knowledge and understanding, and the individual patients’ life circumstances/context being the most prominent issues. Practitioner-level factors identified were practitioners’ speciality, interest and experience in respiratory conditions as the overarching factor that influenced how self-management was understood and practiced. A number of organisational/system-level factors were identified by all practitioners, including inconsistency of referral pathways and the wide variations of different self-management planning tools. Factors affecting self-management of COPD across these three levels need to be tackled equally in order to improve the effectiveness of interventions and to embed and integrate self-management support approaches into routine practice.

## Chronic lung disease: A balanced approach for improved self-management

Better co-ordination between healthcare services, practitioners and patients may help improve self-management for chronic lung disease. Self-management is crucial for patients with chronic obstructive pulmonary disease (COPD), but it can be difficult for healthcare workers to monitor and support patient progress. Oladapo Ogunbayo at Newcastle University, UK, and co-workers conducted interviews with healthcare practitioners to explore perceived barriers to successful self-management of COPD. Three distinct categories emerged; those at patient level, practitioner level and organisational level, the needs of which should be carefully balanced to improved self-management. Patient knowledge and understanding of COPD, alongside individual life circumstances, were often barriers to effective self-care. Those practitioners with specialist respiratory knowledge took a more holistic approach to self-management than their primary care counterparts. A lack of continuity between services and across self-management planning tools presented further barriers.

## Introduction

Chronic Obstructive Pulmonary Disease (COPD) is an increasing cause of mortality and chronic morbidity worldwide. It is currently the fourth leading cause of death and projected to become the third by 2020.^1^ COPD is characterised by persistent respiratory symptoms that include breathlessness, cough and/or sputum production. These symptoms are often due to airflow limitation which is not fully reversible and is likely to result in frequent acute exacerbations of symptoms and a progressive decline of lung function.^2^ Socioeconomic status is strongly associated with the prevalence of COPD, affecting more people from low-income, deprived and educationally disadvantaged communities.^3^ In addition to the social patterning of COPD, people affected are also more likely to have other comorbidities and experience difficulties in many areas of their lives, e.g., financial or family issues, compounding the impact of the disease.^3^

COPD has a significant negative impact on the quality of life of individuals and their families/carers, and also constitutes a substantial social and economic burden on the United Kingdom’s (UK) National Health Service (NHS). People with COPD in the UK have an average of three acute exacerbations per year and these exacerbations are the second biggest cause of unplanned hospital admissions.^4^ The high cost of providing quality care for COPD and other long-term conditions (LTCs) is a challenge for health and social services in the UK and across the world.^5^ The need for a different approach to providing health and social care for LTCs, that includes engaging and activating individuals and their families/carers in aspects of has been recognised.^6^ The complex and varied needs of people with COPD and their social networks however, present a particular challenge for healthcare practitioners.^7^

Self-management has generated a lot of interest in policy and research, and is now a key component of established models of LTC care, including the chronic care model (CCM).^8^ Self-management generally refers to the “individual’s ability to manage the symptoms, treatment, physical and psychosocial consequences and lifestyle changes inherent in living with a chronic condition”.^9^ People living with COPD encounter various practitioners in different disciplines across both primary and secondary care. In addition to management of symptoms, practitioners have successfully supported COPD self-management interventions in such contexts as smoking cessation work,^10^ pulmonary rehabilitation (PR),^11^ and mental health/psychosocial support.^12^ Practitioners also facilitate self-management during patient consultations by providing support with medicines management (inhalers and ‘rescue medications’), managing breathlessness, preventing exacerbations, promoting positive lifestyle changes, and referring/signposting patients to relevant community resources.^13^

Recent research has shown that there is significant heterogeneity in how self-management and self-management interventions for COPD have been defined and operationalised.^14^ A key goal of self-management interventions is sustained positive behaviour change among patients.^13^ Implementing behaviour change techniques (BCTs) that considers a patient’s level of education and literacy, for example, use of visual aids^15^ and ‘teach-back’ techniques^16^ have been recommended to promote patient engagement in self-management.^17^ While the factors affecting general self-management approaches have been explored from the patients’ perspective,^18^ there is little research on practitioners’ perspectives on factors affecting COPD self-management. The few existing studies have either explored practitioners’ perspectives on specific areas of self-management such as PR.^19,20^ COPD guidelines and care recommendations^21–24^ or have focussed on particular practitioners, rather than the wider multidisciplinary team (MDT).

Furthermore, while self-management is embedded within clinical guidelines for COPD,^25^ there are no explicit self-management delivery strategies specified for the different members of the MDT. This qualitative study aimed to explore the views of the MDT to understand their experience of the factors affecting COPD self-management.

## Results

### Participant characteristics

Of the 28 practitioners that were approached, 20 took part in the study. Reasons for non-participation were inability to arrange interview dates/venues and lack of interest. Participants’ mean age was 45 years (range: 26–60 years), 15 were females. All participants were White British and most (15) worked full time. Participants’ mean duration of working within healthcare was 23 years (range: 1–40years). Table 1 below summarises the job role of participants.

**Table 1:** Participants job roles (*n* = 20)

Healthcare team	Job title
Primary care team = 8 (40%)	• General practitioner (GP) = 2
	• Practice nurse = 2
	• Community matron = 2
	• Practice pharmacist = 1
	• Community pharmacist = 1
Specialist respiratory team = 7 (35%)	• Specialist respiratory/COPD practitioners = 6
	• Consultant respiratory physician = 1
Pulmonary rehabilitation team = 5 (25%)	• Respiratory nurse = 1
	• Physiologist = 1
	• Physiotherapist = 1
	• Technical/exercise instructor = 1
	• Occupational therapist (OT) = 1

**Fig. 1:**
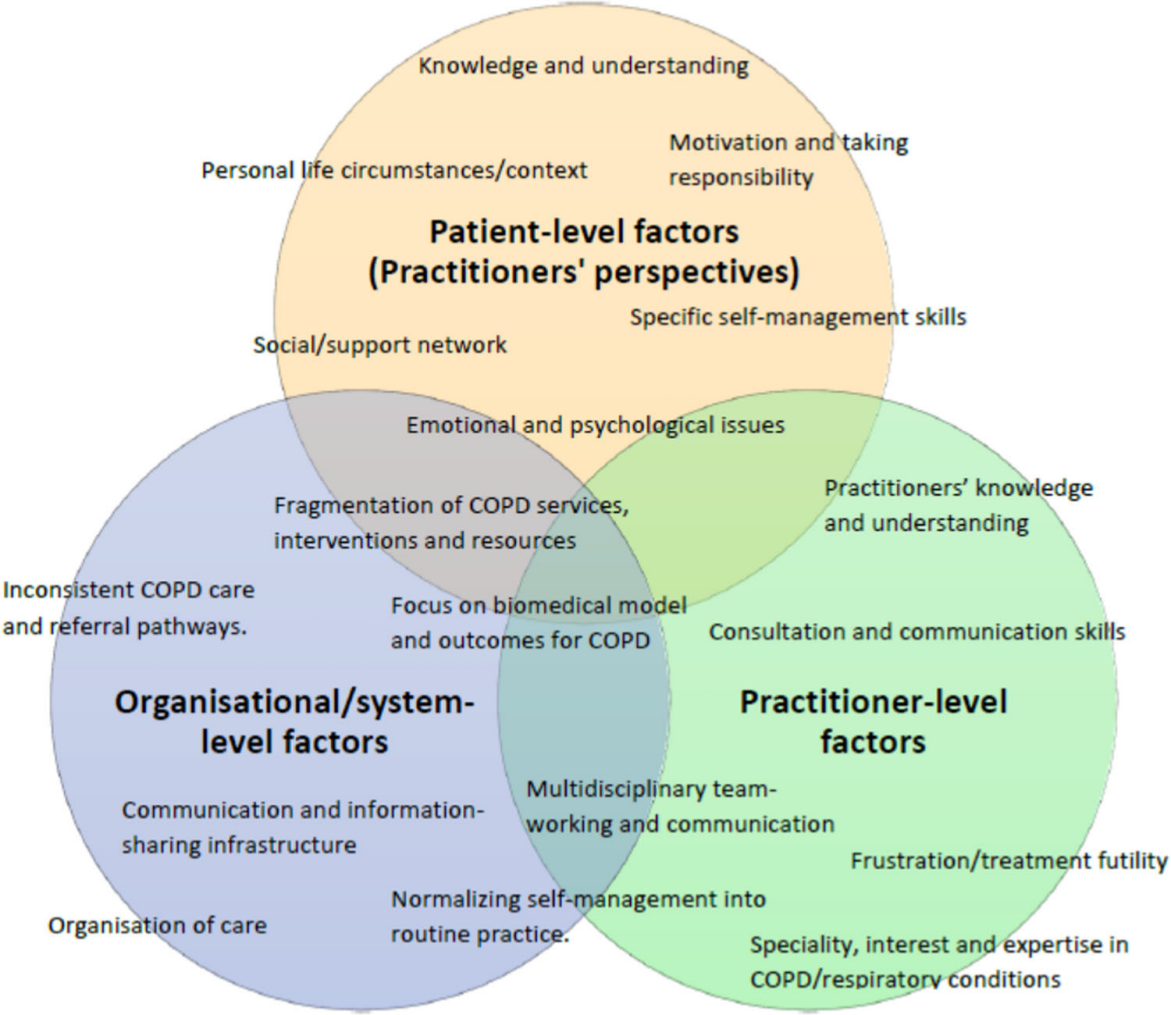
Categories and themes of factors affecting self-management of COPD

Data analysis revealed a range of complex and interrelated factors affecting COPD self-management that were grouped into three broad categories; patient-level, practitioner-level and organisational/system-level factors (Fig. 1). The themes and subthemes that emerged mapped onto these categories.

### Patient-level factors (Practitioner’s perspective)

Practitioners described these as key issues that were associated directly with COPD patients and that influenced individual patient’s attitude and ability to engage in self-management. These factors (Table 2) affected patients in varied ways but were often shaped by their individual life experiences and beliefs.

**Table 2:** Patient-level factors (Practitioners’ perspectives): themes and exemplar subthemes

Themes	Subthemes/exemplar codes
Knowledge and understanding	• COPD aetiology, diagnosis and prognosis
	• COPD trajectory
	• Medicines–inhaler technique and ‘rescue packs’
Motivation and taking responsibility	• Changing behaviour e.g., smoking
	• Uptake of referrals and appointments e.g., PR
	• Managing and maintaining lifestyle
Emotional and psychological issues	• Anxiety and depression
	• Frustration and guilt
	• Self-efficacy and confidence
Specific self-management skills	• Self-monitoring
	• Exacerbation management
	• Problem-solving, goals-setting
Social/support network	• Family and peer support
	• Social isolation
	• Access to support
Personal life circumstances/context	• Comorbidity
	• Living/housing arrangement
	• Socioeconomic conditions
	• Education/literacy

Participants indicated that the patient-level factors, rather than being discrete, overlapped and were intricately connected to each other. The ways that these factors interacted with one another varied, with some factors mutually dependent on each other, while some other factors are a consequence of others. As an example, participants indicated that some patients become socially isolated due to the fear and anxiety of exacerbating their condition when they leave their homes, escalating their lack of interest and involvement in social and community activities that may improve their COPD and quality of life. Similarly, participants indicated that poor patient knowledge and understanding of the aetiology and prognosis of their COPD may reduce their motivation to accept certain behaviour changes (such as quitting smoking) or uptake of a practitioner recommendation (e.g., to attend PR). This reduced motivation may in turn affect how patients engage in specific self-management behaviours such as taking inhalers, self-monitoring and exacerbation management.

And it’s, they don’t understand the progression of their condition and things like that…that’s when your patients do get frustrated, and they lose faith in their inhalers and the people who are trying to help them. “Them inhalers don’t work.”…. **Specialist Respiratory/COPD practitioner**

Patient-level factors described were mostly barriers. Many of the patient-level factors were discussed to varying extents by participants but there was an overwhelming consensus that the personal life circumstances/contexts of individual COPD patients was the key determinant as to whether, and how patients engaged in self-management.

Like I am going to see a lady next week that has got horrendous issues going on. She has got various family problems, money problems, housing problems. Then she has got breathing [problems] and she has got a youngish family. In amongst all that is a person that can’t breathe either. It is pulling that big star together of their lifestyle and trying to work out what is going on. **Respiratory Occupational Therapist**

There was a general consensus that any intervention or approach that targets at least one of these patient-level barriers would be likely to have a cascading effect on other factor(s). For example, most participants believed that improving knowledge and understanding via a targeted and individualised approach to patient education would have the most significant impact on other patient-level factors. Patients’ knowledge and understanding appeared to be the factor that practitioners perceived they could more easily influence amongst other patient-level factors.

Gosh—easiest things. The easiest things depend on individual patients and how they perceive and understanding. I think it is patients’ understanding. If they have got an understanding of the condition—if they know and are able to take things in and absorb things… **Community Matron**

While practitioners acknowledged their role in supporting patients to address the patient-level factors that were barriers to COPD self-management, many suggested that these factors were often within the sphere of control of patients and that patients should take on more responsibility. The key challenge however, was that patients tended to defer most of the responsibility to practitioners.

They have to take a lead on some of it [COPD management]. I think that’s the difficulty with, not all patients, I don’t want to group them all together. The difficulty with a lot of COPD patients is they can sometimes put the onus onto healthcare professionals. **Specialist respiratory nurse**

### Practitioner-level factors

Practitioner-level factors that were identified included those factors directly associated with how the roles, experiences and skills of practitioners influenced how they supported patients to self-manage (Table 3). Most participants did not explicitly view practitioner-level factors as barriers affecting self-management of COPD when compared to patient-level factors.

**Table 3:** Practitioner-level factors: themes and exemplar subthemes

Themes	Subthemes/exemplar codes
Speciality, interest and expertise in COPD/respiratory conditions	• Generalist (Primary care) and specialists (specialist respiratory and PR teams)
	• Specific skillset, e.g., behaviour change, breathing techniques
Practitioners’ knowledge and understanding	• Conceptual understanding of self-management
	• Confirming/delineating COPD diagnosis
Communication/consultation skills	• Behaviour change techniques, e.g., motivational interviewing
	• Patient education vs. self-management education
Multidisciplinary team-working and communication	• Communication and information sharing
	• Multidisciplinary skill-mix
Frustration/treatment futility	• Managing COPD patients’ expectations
	• Dealing with the ‘difficult’ patient
Normalising self-management into routine practice	• Varied self-management planning tools
	• Changing practice/practitioner culture

Practitioners’ specialty, interest and expertise in COPD/respiratory conditions appeared to be the most pertinent factor that determined how they engaged in COPD self-management support. Participants that worked in specialist COPD/respiratory teams and PR teams appeared to be more involved in holistic self-management support.

I really enjoy my job. I really enjoy just having one clientele [COPD patients] to look after….I get the [mix] of seeing patients, sometimes at their very worst, and I get to see them get better in their home environment, and to see them through the progression of being ill, starting treatment, getting better, and assisting them in any other ways ….And I’m constantly looking at, like, their home environment, and whether or not they need any kind of mobility aids, or how they’re managing at home, and problem solving in that kind of sense, still, as well. So I get the whole holistic, kind of, thing with my patients…..I think you get to see the sense of the patient; you get a lot more from the patient, I think, seeing them in their home environment. **Specialist Respiratory/COPD Practitioner**

Participants within primary care teams tended to engage in self-management support from the much narrower perspective of exacerbation management and annual clinical reviews of COPD patients.

The majority of the time when I see patients with COPD is usually when they are perhaps presenting either to me with an exacerbation of COPD or perhaps when they are presented with something else and I am opportunistically taking the chance to review their COPD. Perhaps it is a medication review or if they have come in with a symptom which may be due to the breathing, reviewing their inhalers and management in that respect. Most of the long-term condition organised management, sort of annual reviews are done by the nursing team in the practice… **General Practitioner**

Support provided by the practitioners that viewed self-management as ‘exacerbation management’ focused on providing information about symptom recognition, management and the use of ‘rescue packs’.

I suppose at the moment, when we were taught about self-management, I mainly focused on the self-management like exacerbations at home. I don’t do goal setting, I’ll be honest, for COPD. **Practice Nurse**

Many participants recognised the need for improved communication with COPD patients and enhanced consultation skills in areas such as the use of BCTs (e.g., Cognitive Behaviour Therapy (CBT), motivational interviewing); specific self-management support skills (e.g., collaborative action-planning and goal-setting); dealing with patients’ psychological and emotional issues; and the use of a consistent referral pathway (e.g., social prescribing).

I know some of the nurses are doing their introduction to CBT [cognitive behavioural therapy]. I think it is quite important that we all have that ability to be able to challenge somebody’s thought process. It doesn’t matter whether you are a nurse going in or an OT going in or a physio or a doctor, you still need to be able to sing from the same song sheet. If you all have a little bit of knowledge about that skill to be able to say to a respiratory patient, “You will be fine. Let’s talk about it. Let’s explore this a little bit further”. I think you all need to have that bit. **Respiratory Occupational Therapist**

However, some participants indicated that they already applied some form of BCTs in their interaction with patients even though this may not follow a structured or systematic format.

You probably do [behavioural counselling/motivational interviewing] but without formally knowing that that’s what you are doing…. It’s trying to work with the patient, trying to understand their needs. Getting onto their level because their level is going to be different with every patient. Try and find out what their needs are, what their education needs are and then working with that. **Specialist Respiratory/COPD practitioner**

### Organisational/system-level factors

Organisational/system-level factors (Table 4) were felt to operate at a higher-level than patient- and practitioner-level factors and included the organisational processes and commissioning arrangement that influenced self-management. For example, the lack of continuity in some services and interventions available to COPD patients, for example PR, were perceived as being intrinsic to the ways that these services were commissioned.

Another thing which is frustrating for us, the programme (PR) only lasts six weeks and it is a rolling programme so we have just discharged a lot of people. We feel like at the time, especially in this venue, we get a class put together you start completing people….We would like a class and we would like longer than six weeks maybe. It is the way it is commissioned by CCGs. **Pulmonary Rehabilitation Practitioner (Instructor)**

**Table 4:** Organisational/system-level factors: themes and exemplar subthemes

Themes	Subthemes/exemplar codes
Fragmentation of COPD services, interventions and resources	• Awareness and access to resources
	• ‘Postcode’ lottery
Focus on biomedical model and outcomes for COPD	• ‘Over-medicalisation’ of COPD care
	• Incentives, e.g., QOF targets
Organisation of care	• Reactive system
	• Convoluted care pathway/referral systems
Communication and information-sharing infrastructure	• Continuity of care
	• Slow system
Inconsistent COPD care and referral pathways.	• Varied self-management planning tools
	• Local variations

Organisational/system-level factors varied, depending on the healthcare teams that participants worked within. On the whole, factors relating to inconsistency of referral pathways (e.g., to PR or to specialist teams) and the wide variations of different self-management planning tools (e.g., British Lungs Foundation self-management plans) were acknowledged by almost all participants as a cross-cutting factor that affected how they engaged in self-management of COPD.

It would be really nice that we all use the same self-management plan. That would make things a whole lot easier, the hospitals and us. That is a big thing, and it’s got to be a simple plan because we haven’t got hours to sit and go through it. **Practice Nurse**

Some of the organisational/system-level factors were more evident among participants working within specific healthcare teams, for example, the systematic focus on the biomedical approach to the management of COPD within primary care teams, where the use of ‘rescue medications’ were prioritised, influenced how some practitioners provided self-management support and planning.

It [self-management plan] says if your symptoms are well-controlled, use your normal medication, if you become more breathless you increase your Salbutamol, then if you start coughing more phlegm up, the phlegm changes colour, you’re more short of breath, start your rescue pack….I would because we aim to give everybody a rescue pack…**General Practitioner**

Similarly, participants working within PR teams expressed their frustration with referrals and uptake of PR services by patients despite its wide availability and awareness of the service. Participants here indicated that referrals to the service varied across different geographical areas and suggested an organisational approach that promoted better communication and integration of the service into routine patient care.

Our role is to provide a service, Pulmonary Rehab, but we’re doing more than that. We’re going out with leaflets. We’re visiting doctor’s surgeries. We’re visiting the hospital. We’ve sat and talked in front of consultants. I don’t know what it is with some people that there seems to be a barrier…. It’s hard to understand as well because you know the patients are out there. These classes should be full. That’s what we find the most frustrating….**Pulmonary Rehabilitation/Respiratory Nurse**

Participants also indicated that organisational/system-level factors could concurrently affect both COPD patients and practitioners, for example, in terms of awareness, availability and access to self-management resources. Some participants indicated that many COPD patients may be confined to a ‘postcode lottery’ in the care they received based on their geographical location leading to inconsistencies and inequalities in the care provided.

…There is a postcode lottery whether you get steroids and a rescue pack. That seems to be the in thing. “I haven’t got steroids. I haven’t got antibiotics”. There is a mixed bag about that. **Respiratory Occupational Therapist**

Likewise, a reactive system inherent with providing referrals to high value interventions like PR where practitioners only refer patients who have experienced a critical incident (frequent exacerbation or hospital admission), and patients who otherwise may have benefited from this service are missed.

On our computer, we’ll have a flash up score that if you’ve gone through the score with them [the patient] and it is breathless, it does pop up to say, “Please offer…” And it does every time until you say yes or no, you’re going to. But I would say, yes, it is probably the ones that have had admissions to hospital that are more referred than perhaps in general practice…. I think we could perhaps be doing more to offer them… It would probably, as I say, be better for them to- Before they got really poorly and needed admissions to hospital if we could help them, or find ways to help them before they got to that stage, it might prevent hospital admissions. **Practice Nurse**

## Discussion

### Main Findings

Our analysis reveals a complex range of factors that affects self-management of COPD at different levels. Categorising these factors into three levels, patient-, practitioner- and organisational/system-levels, helped to unpack the nuances in the themes that emerged. Among the patient-level factors identified in this study, the individual life circumstances/context of COPD patients was the key factor described by practitioners that determined whether, and how patients engaged in self-management. There was, however, recognition of the challenges of providing holistic and personalised support, particularly among practitioners in primary care, who prioritised addressing patients’ knowledge and understanding over other factors. Other practitioners (specialist respiratory and PR teams) that would otherwise be able to provide these more holistic patient-centred support are often limited by the commissioning arrangement of their service (e.g., early hospital discharge and readmission prevention service and PR service), where they often tend to see patients episodically and usually after a critical incident.

Practitioner’s specialty, experience and interest in COPD/respiratory diseases was the prominent practitioner-level factor that influenced how COPD self-management was supported in practice. The more specialist practitioners (specialist respiratory and PR) appeared to have a better, more holistic grasp of self-management, whereas, the generalist practitioners (primary care) engaged in COPD self-management from a narrower perspective (mainly exacerbation management). Whilst this specialist/generalist divide is an understandable feature of the health system, there is a need to develop ways of providing a more consistent self-management message and strengthen existing pathways for the benefit of the patient. The organisational-level factors highlighted were more specific to the practice settings of the different practitioners but pointed to the need of a more joined-up communication and coordinated system to deliver a comprehensive and consistent self-management package for patients with COPD.

### Interpretation of findings in relation to previous work

The factors affecting self-management of COPD have been conceptualised as being along a spectrum of barriers and facilitators.^18^ The patient-level and organisational/system-level factors in our study were mostly located at the ‘barriers’ end of the spectrum in comparison to practitioner-level factors. While practitioners perspectives of patient-level factors are consistent with what is known in the literature about barriers to self-management,^22–24,26^ the practitioner- and organisational/system-level factors provide new insights into the operational barriers that could hinder the implementation of COPD self-management interventions. Most COPD self-management interventions focus their attention and investment in addressing the key patient-level factors while practitioner-level and organisational-level factors are given less attention.^27^

The alignment of the beliefs and priorities of both patients and practitioners are essential pillars in the delivery of self-management and care for people with COPD and other LTCs.^28,29^ A large body of review literature has reported on factors affecting self-management of COPD from the perspectives of patients (and their families)^18,30^ but few studies have explored the perspectives of practitioners.^31,32^ Among the few practitioner studies, the perspectives of the wider MDT is limited. Hence, considering the perspectives of the various MDTs working across different settings is crucial to understanding the delivery and implementation of care in such a complex condition as COPD.^33,34^

Our study highlighted the differences in how practitioners working in different settings approached self-management support of COPD. The need for better cooperation, communication and information-sharing among the different primary care and secondary care practitioners in the care of COPD has previously been reported.^35^ In addition, practitioners in this study described variability in access and uptake of interventions that promote COPD self-management across different geographical areas, which has potential to widen health and care inequalities.^36^ Practitioners however recognised the need for increased practitioner knowledge and awareness of local/community services and interventions that promote self-management. With the right organisational support systems in place, the proactive use of enhanced referral pathways and social prescribing strategies may help to tackle this.^36,37^ Furthermore, models such as the CCM^8^ and the House of Care^28^ prescribe changes to organisational processes (e.g., integrated IT systems and responsive commissioning) as a way of addressing these and other organisational/system level factors.

The management of COPD by practitioners is often ‘over-medicalised’ with primary focus on the use of inhalers and rescue medications, whereas self-management interventions is not often given adequate attention.^35^ Patient education to address the gap in knowledge and understanding appeared to be the predominant self-management support strategy adopted by practitioners in this study. While patient education is a key aspect of self-management support, this alone is insufficient in changing behaviour as it requires individualised support that incorporates BCTs.^38^ Some authors have made a distinction between patient education and self-management education approaches, where the former is viewed as biomedical, generic and didactic, and the later patient-centred, holistic and tailored to individual patient’s needs.^39^ Studies have shown that practitioners, particularly in primary care often prioritised patient education approaches during routine consultation with patients.^40,41^ Practitioners however recognised the challenge of providing holistic and personalised support, particularly among primary care practitioners, who are often inherently constrained by time and pressure to meet performance targets.^42^

A recent qualitative meta-synthesis of clinicians’ perspectives on their roles in self-management more generally identified factors related to negotiating control with patients (didactic model vs. patient-centred approaches) and difficulties of embedding self-management into routine clinical practice.^31^ In addition, studies have found a dichotomy of self-management priorities between patients and practitioners.^32^ For example, where goalsetting and motivation were considered a requisite for successful self-management by practitioners, patients and family members did not identify this as an important self-management outcome. Instead, skills in managing emotions and stress were considered more important to maintain health and wellbeing.^32^ General practice nurses described key factors that affected self-management as poor knowledge of COPD and aspects of self-management amongst nurses, competing demands, poor team working and lack of multidisciplinary support.^43,44^ Primary care doctors, nurses and allied health practitioners working across primary, community and secondary settings have also identified barriers relating to poor understanding and awareness of COPD (e.g., delineating between COPD and asthma diagnosis), limited time, lack of awareness of educational and learning needs and expectations about patients’ self-management capabilities.^22,26^ Similar findings have also been reported in studies in other LTCs.^45,46^ Our study supports these findings and has brought the perspectives of these different practitioners together, further highlighting the challenges of COPD care integration across different practitioner teams.

Organisational/system level factors relating to communication, information-sharing and making referrals (e.g., for PR) and the practical difficulties that patients face have been reported in some studies.^19^ The first national primary care COPD audit in England and Wales found that the use of triple therapy interventions, which were expensive and potentially less effective interventions, were often prioritised over high value interventions such as PR, flu vaccination, and smoking cessation advice.^47^ Our study has highlighted patient-level, practitioner-level and organisational/system-level factors that potentially hinder practitioners from delivering these high value COPD self-management interventions. These findings point to a whole-systems approach to delivering self-management support of COPD with components that operates at patient, practitioner and organisational level.^48^ Implementation studies that have explored a whole-systems approach in COPD are sparse,^45^ but evidence from other LTCs like asthma,^49^ and diabetes ^50^ showed significant improvements in clinical and health outcomes, and reduced hospital admission.^45,49^

### Strengths and limitations

A key strength of this study was the inclusion of a range of multidisciplinary healthcare team members from both primary and secondary care involved in the care of COPD, which has provided a more nuanced understanding of the factors affecting self-management of COPD. However, due to the relatively small numbers of participants from each group, the transferability of the findings should be treated with caution. The involvement of two researchers in the data collection and analysis improved rigour and strengthened the study’s analysis and findings. Respondent validation of the final coding framework was undertaken via participatory workshops with practitioners and this further strengthened the credibility of the findings. While participants were recruited purposively to achieve a representative sample, some practitioners such as mental health practitioners and healthcare assistants were not included. This may warrant further investigation as some of these practitioners are also involved in delivering specific self-management interventions, e.g., CBT and social prescribing. Furthermore, participants were recruited based on self-selection, which may indicate that participants were already interested in the topic area and may have provided socially desirable perspectives.

### Implications for future research, policy and practice

This study highlights the need for multilevel strategies that tackle the factors that hinder self-management of COPD from the patient, practitioner and organisational perspectives. In order to ensure the effectiveness of interventions and to enhance the integration of self-management support approaches into routine practice, the barriers identified across these three levels need to be tackled equally. Effing et al.^17^ proposed a conceptual description of COPD self-management interventions that mostly considers factors operating at the patient- and practitioner-level, but not the organisational level. There is currently limited research of how organisational/system-level factors affect self-management of COPD and the perspectives of key stakeholders such as policy-makers and commissioners of health services is evidently absent in the literature.^32^ This is an important area of research that could potentially, be the missing link to improving the implementation of COPD self-management interventions. Findings from implementation studies in disease areas such as asthma and diabetes could be incorporated in the design of whole-systems approach to self-management of COPD.^45^

Primary care practitioners recognised the challenge of providing holistic support for COPD within a clinically-focussed GP environment. Holistic self-management support for COPD may be better provided outside the clinical environment, for example in patients’ homes. While other practitioners such as specialist respiratory teams are able to provide this holistic support, these are often for a short durations. This study supports the case for emerging social prescribing models that make use of health navigators/link workers to provide long-term comprehensive self-management support within patients’ homes.^37^ Furthermore, primary care practitioners may benefit from joint/inter-professional education and networking with other members of the MDT to provide opportunities to share experiences and improve skills in areas such as the use of BCTs and provision of holistic COPD self-management support. In addition to promoting better communication and teamwork across different practitioner teams, a move towards models of care such as the Year of Care^51^ that embeds care and support planning in primary care consultations for COPD may prove beneficial. Another approach may be to embed specialist COPD practitioners within primary care and community teams, for example in diabetes, to ensure continuity of self-management along the trajectory that COPD patients go through.

## Conclusion

Self-management of COPD is an essential component of the care provided by practitioners. An understanding of barriers and enabling factors from the perspectives of practitioners is crucial to the provision of high quality healthcare. This study identified a number of patient-level, practitioner-level and organisational/system-level factors that should be given balanced attention if any COPD self-management intervention is to be successfully implemented. Different members of the multidisciplinary healthcare team involved in COPD care approach self-management from different perspectives and this variation has important implications for the provision of a consistent self-management package for COPD.

## Methods

### Study design

A qualitative approach that employed semi-structured interviews, underpinned by the philosophical stance of hermeneutic phenomenology,^52^ to explore participants’ subjective accounts while also acknowledging the roles and preconceptions of the researchers. The study received the National Health Service (NHS) Research Ethics Committee (REC) and Research and Development (R&D) approval (REC Reference 15/NW/0951) and the methods were performed in accordance with relevant guidelines and regulations.

### Study setting

The study was based in Northeast England which has rates of COPD higher than the national average in terms of mortality, prevalence, health outcomes and health service utilisations. (https://statistics.blf.org.uk/copd).

### Study population and sampling

Participants were practitioners involved in the care of COPD across both primary and secondary care settings. Participants were recruited by purposive sampling to achieve maximal variation^53^ in job role, i.e., primary care, specialist respiratory and PR teams. Participant recruitment and sampling continued until theoretical data saturation was achieved.^54^

### Recruitment strategy

Potential participants were initially identified and recruited by clinical members of the research team and other key contacts who were practitioners. Further recruitment was undertaken by attending practitioner training and engagement events, as well as via snowballing^55^ to achieve a good variation of perspectives. Potential participants were invited to take part in the interviews via emails (with the study’s information sheet attached), and were followed up via telephone calls when necessary. Suitable dates and venues were arranged with participants that agreed to be interviewed.

### Data collection

Recruitment and interviewing of participants took place between February and July 2016. Most interviews were conducted face-to-face, and one on the telephone. Interviews took place mostly at the participants’ places of work or other agreed location (e.g., Café, university). The interviews were directed with a topic guide (Table 5) that was developed from the literature and evolved iteratively as the interviews progressed. The topic guide used open-ended questions with prompts and cues to explore the topic area. All participants provided written (signed) and verbal consent before the interviews commenced. Interviews were audio-recorded and lasted for approximately 90 min.

**Table 5:** Practitioner topic guide

Broad topic area	Specific question area and probes
Background	Introduction and experience
	• Education, work history, job role, area of practice/specialty
	• Special interest in COPD?
	‘Typical’ day at work
	• Patient type—working with COPD and/or other LTCs
	• Interaction with other HCPs—GPs, nurses, hospitals, specialists, PR, etc
Understanding of the concepts of ‘self-management’ and self-management support	Describe the term ‘self-management’
	• Who and what is involved?
	• How do COPD patients ‘self-manage’?
	Based on how you described ‘self-management’, how do you provide support?
	• Generic (checklist) vs. personal approach?
	• Is this different for other conditions?
Self-management support for COPD	Self-management/self-monitoring support
	• Stable COPD
	• Acute exacerbations
	Specific COPD self-management interventions
	• Medical (medications/inhalers, ‘rescue packs’) and lifestyle (smoking, physical activity, diet)
	• Mental/psychological health
	• Referrals/signposting to PR, community teams, CBT
	Self-management planning
	• COPD specific plans
	• Action planning, goal setting and follow-up
	• Involving patients in shared decision-making during consultation?
Strategies for implementing self-management support of COPD	Using specific examples, can you describe strategies on;
	• Managing patient confidence/self-efficacy
	• Motivation to engage in self-management
	• Dealing with ‘difficult’ patients
	• Organisational/practical support
	• Managing resources and services
	In your current role/practice, how do you think COPD self-management can be improved?
	• What currently works well?
	• What doesn’t?
	Are you confident in your ability to routinely deliver COPD self-management support?
	• Use of any behaviour change approach?
	• Learning needs/training?
	• Multidisciplinary approach?—roles for other team members
	• Continuity of care?—e.g., transiting from hospitals to community care
	Any concluding thoughts/comments/questions?

### Data analysis

Data collection and analysis were undertaken by two researchers on the project (SR and OO). Audio files were stored digitally on a secure computer network at the researchers’ University office. The interviews were transcribed verbatim using a professional service. Interview transcripts were anonymised and were transferred to the QSR NVIVO software (version 11) to help manage and retrieve the data. Data analysis employed an interpretative thematic approach.^56^ The two researchers initially analysed a sample of the transcripts separately, discussed the initial themes and agreed on a preliminary coding framework initially based on the topic guide. This coding framework was then used to analyse the rest of the transcripts. Analysis was a cyclical and iterative process that initially involved data familiarisation (initial reading of the interview transcripts) which generated themes and subthemes. The data analysis process continued with a close reading and reading of the texts with further refinements, connections and relationships made between the emergent subthemes, themes and categories. The coding and interpretation process was continuous and extensive and led to the emergence of a final coding framework consisting of three broad categories and number of themes and subthemes (Tables 2, 3 and 4). This was agreed upon mainly by two researchers and other members of the research team, and were cross-checked against the interview transcripts. Respondent validation of the final coding framework was undertaken during two participatory workshops with practitioners that was conducted as part of the wider research project. These participatory workshops included 11 practitioners, some of whom were involved in the interviews.

### Data availability statement

The datasets generated during and/or analysed during the current study are available from the corresponding author on reasonable request.
